# The combination of allicin with domiphen is effective against microbial biofilm formation

**DOI:** 10.3389/fmicb.2024.1341316

**Published:** 2024-05-30

**Authors:** Shang Li, Yutong Wang, Geweirong Xu, Yuqing Xu, Cuiyan Fu, Quanlin Zhao, Linjie Xu, Xinzhou Jia, Yumeng Zhang, Yi Liu, Jiaju Qiao

**Affiliations:** ^1^Department of Biotechnology, School of Life Sciences, Xuzhou Medical University, Xuzhou, China; ^2^Department of Biophysics, School of Life Sciences, Xuzhou Medical University, Xuzhou, Jiangsu, China; ^3^School of Pharmacy, Xuzhou Medical University, Xuzhou, Jiangsu, China; ^4^Jiangsu Key Laboratory of New Drug Research and Clinical Pharmacy, Xuzhou Medical University, Xuzhou, Jiangsu, China

**Keywords:** food pathogen, biofilm formation, drug combination, natural product, cross contamination

## Abstract

**Background:**

Microorganisms in biofilms are particularly difficult to control because of their increased survival and antibiotic resistance. Allicin and domiphen were employed to inhibit the microbial growth and biofilm formation of *Staphylococcus aureus*, *Escherichia coli*, and *Candida albicans* strains.

**Methods:**

Broth microdilution method and checkerboard assay were conducted to determine the efficacy of allicin combined with domiphen against *S. aureus*, *E. coli*, and *C. albicans*. Microbial biofilm formation was measured using the crystal violet staining method and fluorescence microscopy. And the total viable count of the biofilm cells on material surface after the treatment with antimicrobial reagents was calculated with the plate count technique.

**Results:**

The two drugs showed synergistic effects against the pathogens with a fractional bactericidal concentration of less than 0.38. The combination of 64 μg/mL allicin with 1 μg/mL domiphen dispersed over 50% of the biofilm mass of *S. aureus*, *E. coli*, and *C. albicans*. In addition, the drug combination reduced the total viable counts of *E. coli* and *C. albicans* biofilm cells on stainless steel and polyethylene surfaces by more than 10^2^ CFU/mL.

**Conclusion:**

The combination of allicin and domiphen is an effective strategy for efficiently decreasing biofilms formation on various industrial materials surfaces.

## 1 Introduction

Bacterial and fungal species (Lohse et al., 2017) form biofilms as a survival strategy (Teschler et al., 2015). Biofilm formation enhances the bacterial resistance to constantly changing and hostile environmental conditions (Mevo et al., 2021). Biofilms, which are communities of microbial cells embedded in extracellular polymeric substances, can form on food contact surfaces, leading to hygiene concerns (Guo et al., 2021). Pathogenic biofilms can cause cross-contamination of work surfaces (stainless steel, plastic, and silicon rubber) and food surfaces (chicken skin and eggshells) (Roy et al., 2023). Biofilm formation on piping, equipment, or heat exchanger surfaces can cause food safety issues, decrease production efficiency, lead to economic loss, and reduce consumer trust (Alonso et al., 2023). Moreover, bacterial biofilms on the surfaces of indwelling medical and dental devices make the treatment of infections increasingly difficult (Linklater et al., 2021). Biofilms formation within the host have been implicated in diseases such as cystic fibrosis, urinary tract infections, and endocarditis (Guo et al., 2022). The negative impacts of microbial biofilms are incalculable, resulting in an annual loss of millions of US dollars (Cattò and Cappitelli, 2019).

*Escherichia coli* biofilms increased their resistance to environmental stress and survive sanitization treatments (Serra and Hengge, 2021). Outbreaks of *E. coli* pose a significant threat to human food safety. In fact, *E. coli* is the main cause of acute diarrhea in children in most African, Asian, and Latin American countries (Shivaprasad et al., 2021). *S. aureus* is a prevalent human pathogen (Tomlinson et al., 2021), known for its frequent association with biofilm-related infections of indwelling medical devices (Schilcher and Horswill, 2020). Moreover, it is commonly linked to foodborne illnesses in the meat and dairy industries (Guo et al., 2021). *C. albicans*, a fungal pathogen, can form biofilms on the surfaces of teeth (Du et al., 2020), host tissues (Fan et al., 2022), and medical devices (Lohse et al., 2017). Therefore, measures to control the formation of microbial biofilms and reduce the risk of contamination are urgently required.

The minimum inhibitory concentration (MIC) of bacteria in biofilms maybe 10–1,000 times higher than that of planktonic bacteria (Li et al., 2021). Methods for improving the efficiency of antimicrobials in biofilm removal are urgently required. Several new strategies have been suggested to prevent biofilm formation. Phytochemicals have shown excellent performance in preventing biofilm formation and killing resident bacteria within biofilms (Uddin Mahamud et al., 2022). Natural products (Rossi et al., 2020) have been extensively used as eco-friendly antibiofilm agents to minimize the side effects of conventional methods (Guo et al., 2021) on human health and the environment. Plant extracts containing terpenoids, polyphenols, and thiols are widely used to promote antibiotic-free treatment in the post-antibiotic era (Rossi et al., 2020). Allicin is a natural plant extract with broad-spectrum antibacterial properties (Wüllner et al., 2019). Allicin disrupts the balance between the sulfhydryl and redox groups, affects protein homeostasis and cell membrane integrity, and inhibits pathogen infection (Gutiérrez-del-Río et al., 2018). In addition, allicin decreases *Pseudomonas aeruginosa* biofilm formation by reducing adhesion and extracellular polysaccharide production (Bhatwalkar et al., 2021). Domiphen as an antiseptic, antibacterial, and disinfectant agent, is widely used in the pharmaceutical and cosmetic industries (Fumagalli et al., 2018). Domiphen is a surfactant, the hydrophobic portion typically anchored to the fouling surface, and the hydrophilic head is embedded in the bulk aqueous phase (Sun et al., 2018). The biofilm matrix is dispersed from the contact surface into the solvent after surfactant treatment to avoid repeated contamination.

Optimizing the dosage of existing antibacterial agents is crucial for improving their anti-infective effects and reducing antibiotic resistance (Wu et al., 2015). Treating biofilms using a comprehensive approach is more effective than using a single antimicrobial agent (Wu et al., 2015). In the food industry, combining natural antibacterial ingredients can reduce the number of antimicrobial agents, balance antibacterial activity, and increase economic benefits (Li et al., 2023). A combination of antimicrobial agents is recommended for the effective treatment of microbial biofilm contamination.

In this study, the allicin and domiphen combination was used for the first time to control biofilm formation by pathogenic microorganisms. The drug combination was applied to polyethylene and stainless-steel surfaces, which are commonly used in the food and medical device industries. This drug combination reduced the dosage of the two drugs, resulted in an improved anti-biofilm effect, and avoided secondary pollution caused by the residual biofilm matrix. This suggests that the combination of allicin and domiphen is a promising strategy for biofilm scavenging.

## 2 Materials and methods

### 2.1 Materials

*Staphylococcus aureus* ATCC 6538, *E. coli* 8099 and *C. albicans* ATCC 10231 were purchased from the Chinese Center of Industrial Culture Collection. Allicin was obtained from TransGen (Beijing, China). Domiphen was purchased from ABM (Nanjing, China). Mueller-Hinton Broth (MHB) was purchased from Hope Bio-Technology (Qingdao, China). Crystal violet staining solution was purchased from KEYGEN Bio-Technology (Jiangsu, China). The fluorescent stain SYTO9 and propidium iodide (PI) was obtained from KeyGen (Nanjing, China).

### 2.2 Antimicrobial susceptibility

Broth microdilution method was performed according to the Clinical and Laboratory Standards Institute (CLSI) guidelines (Qiao et al., 2020). *S. aureus* ATCC 6538, *E. coli* 8099, and *C. albicans* ATCC 10231 were cultured at 37°C for 10 h until the log phase. The bacterial cell concentrations were adjusted to an OD_595_ of 0.5 (10^8^ CFU/mL), and diluted to 10^5^ CFU/mL using MHB medium. The maximum concentration of allicin was 1,024 μg/mL and that of domiphen was 128 μg/mL. These reagents were diluted by the 2-fold with MHB. The minimum inhibitory concentration (MIC) was read after incubation at 37°C for 18 h. The cells mixed with MIC, 2MIC, 2^2^ MIC, 2^3^ MIC, 2^4^ MIC, and 2^5^ MIC antibiotics were plated on Luria-Bertani agar at 37°C for 24 h. If ten or fewer colonies resulted from the subculture, the minimum bactericidal concentration (MBC) was recorded.

### 2.3 Antibacterial activity of allicin combined with domiphen

The efficacy of allicin combined with domiphen was evaluated using the checkerboard assay. Allicin (16, 32, 64, 128, and 256 μg/mL) and domiphen (1, 2, 4, 8, and 16 μg/mL) domiphen were added to 96-well microtiter plates. The final concentrations of *S. aureus* ATCC 6538, *E. coli* 8099, and *C. albicans* ATCC 10231 cells were adjusted to 10^5^ CFU/mL. After incubation at 37°C for 18 h, the MIC of allicin in combination with domiphen was determined. The MBC was similar to that described in section 2.2.

The fractional inhibitory concentration (FIC) and fractional bactericidal concentration (FBC) indices were obtained by calculating the MIC and MBC, respectively (Tamang et al., 2022). The FIC index was calculated as follows:

FIC = MIC_A_’ /MIC_A_ + MIC_B_’/MIC_B_,

where MIC_A_ and MIC_B_ are the MICs of agent A and agent B alone, respectively, and MIC’ is the MIC of agents A and B in combination. Similarly, FBC was calculated as follows:

MBC_A_’/MBC_A_ + MBC_B_’/MBC_B_,

where MBC_A_ and MBC_B_ are the MBCs of agent A and agent B alone, respectively, and MBC’ is the MBC of agents A and B in combination.

### 2.4 Inhibition of biofilm formation

Microbial biofilm formation was cultured in a nutrient broth (NB) medium and measured using the crystal violet staining method (O'Toole et al., 1999). Briefly, *S. aureus* ATCC 6538, *E. coli* 8099, and *C. albicans* ATCC 10231 cells were cultured at 37°C for 8 h. Every sample (10^6^ CFU/mL) was added to 96-well microtiter plates at 200 μL per well and repeated 6 wells. After incubation at 37°C for 1 h, the culture medium was discarded, and cells were washed with PBS (pH 7.2). Biofilms were then cultured in NB medium with different concentrations of allicin (32, 64, 128, 256, and 512 μg/mL) or domiphen (1, 2, 4, 8, 16, and 32 μg/mL). After incubation at 37°C for 24 h, the culture medium was discarded, and the biofilms were washed three times with sterile water and stained with 200 μL crystal violet for 30 min. The biofilms were rinsed with sterile water to remove the dye. Then, 200 μL of 95% ethanol was added to each well and placed at room temperature for 20 min. Finally, OD_595_ values were measured using a microplate reader to determine the formation of biofilm. Minimum biofilm-eliminating concentrations (MBECs) of antimicrobial agents were recorded as 50% biofilm removal (MBEC_50_) (Tetz et al., 2016).

### 2.5 Quantification of biofilm formation and eradication

#### 2.5.1 Inhibition of biofilm formation

The checkerboard assay was used to analyze the anti-biofilm effects of the drug combination against *S. aureus* ATCC 6538, *E. coli* 8099, and *C. albicans* ATCC 10231. The concentration of allicin was 32, 64, 128, 256, and 512 μg/mL. The concentration of domiphen was 1, 2, 4, 8, 16, and 32 μg/mL. Drug samples (100 μL) and microbial suspension (100 μL; 106 CFU/mL) were mixed in 96-well microtiter plates each well. The culture medium was discarded after incubating at 37°C for 24 h (David Sacks et al., 2018). The subsequent treatment was similar to that described in section 2.4. The fractional biofilm eradication concentration (FBEC) index was obtained by calculating MBEC (Dall et al., 2018). The FBEC index was calculated as follows:

FBEC = MBEC_A_’ / MBEC_A_+ MBEC_B_’/ MBEC_B_,

where MBEC _A_ and MBEC _B_ are the MBECs of agents A and agent B alone, respectively, and MBEC’ is the MBEC of agents A and B in combination. The MBEC_50_ of drug combination were recorded, too.

#### 2.5.2 Removal of biofilm on the surface of stainless steel

*Staphylococcus aureus* ATCC 6538, *E. coli* 8099, and *C. albicans* ATCC 10231 were cultivated to the mid-log phase at 37°C. The microbial suspension (2 mL, 10^6^ CFU/mL) was added to a 6-well plate pre-placed with a steel plate. The medium was discarded after incubating at 37°C for 12 h and washed three times with sterile water. To remove the biofilm, 2.5 mL of allicin (64 μg/mL), domiphen (1 μg/mL), or a combination of allicin (64 μg/mL) and domiphen (1 μg/mL) was added for 4 h. Phosphate-buffered saline was added as the control group. The samples were rinsed three times with sterile water, immersed into 20 mL of sterile PBS (in the 50 mL falcon tubes), and sonicated at 20 kHz for 10 min (Ultrasonic Cleaner KQ3200, China). After sonication, 1 mL of the microbial suspension was serially diluted, plated on Luria-Bertani agar, and incubated at 37°C for 18 h. Finally, the total viable count of the biofilm cells after the treatment with antimicrobial reagents was calculated.

#### 2.5.3 Removal of biofilm on the surface of polyethylene material

*Staphylococcus aureus* ATCC 6538, *E. coli* 8099, and *C. albicans* ATCC 10231 were cultured at 37°C to mid-log phase. Then, 2 mL of microbial suspension (10^6^ CFU/mL) was added to a 6-well cell culture plate (35 mm diameter, 2 mm height). The treatment was similar to that described in section 2.5.2.

#### 2.5.4 Analysis of biofilm by fluorescence microscopy

Fluorescence microscopy was used to evaluate microbial viability after treatment with allicin and domiphen. The microbial biofilm was cultured for 48 h, washed to remove planktonic cells, and treated with a combination of allicin and domiphen for 4 h. The biofilms were stained using a mixture of 200 μL 10 μM SYTO9 and 20 μM PI solution at room temperature in the dark for 30 min. The stained biofilm was then scanned using an Olympus IX73 fluorescence microscope (Kurz et al., 2021). ImageJ software was used to analyze fluorescence intensity.

### 2.6 Statistical analysis

Statistical differences were evaluated using IBM SPSS Statistics 20 (New York, USA), and all experiments were repeated at least three times. One-way analysis of variance was used for every figure, and the means were compared using Tukey’s multiple-range tests. Values are presented as means ± standard deviations. Statistical significance was set at *p* < 0.05, * indicates *p* < 0.05, and ** indicates *p* < 0.01, compared to the control group. The results were plotted using Origin 8.5 software.

## 3 Results

### 3.1 The combined effect of allicin and domiphen against microorganisms

Broth microdilution method was conducted to determine the minimum inhibitory concentration (MIC) of allicin and domiphen against *S. aureus*, *E. coli*, and *C. albicans*. Allicin exhibited similar inhibitory effects on all three strains, with a MIC value of 128 μg/mL. Domiphen showed inhibitory effects against *E. coli* and *C. albicans* strains with MIC values of 2–4 μg/mL and 4 μg/mL, respectively. It also exhibited an inhibitory effect on *S. aureus* with a MIC value of 2 μg/mL (Table 1). The minimum bactericidal concentration (MBC) was determined using the dilution coating plate method. Allicin exhibited bactericidal effects against all three strains, with MBC values of 256 μg/mL, 256 μg/mL, and 512 μg/mL, respectively. Similarly, domiphen exhibited bactericidal effects against all three strains, with MBC values of 8 μg/mL, 4 μg/mL, and 8 μg/mL, respectively.

**Table 1:** Antibacterial effect of allicin or domiphen.

Strains	MIC (μg/mL)		MIC’ (μg/mL)		FIC	MBC (μg/mL)		MBC’ (μg/mL)		FBC
	Allicin	Domiphen	Allicin	Domiphen		Allicin	Domiphen	Allicin	Domiphen	
*S. aureus* ATCC 6538	128	2	64	1/2	0.75	256	4	64	1/2	0.38
*E. coli* 8099	128	2–4	32	1	0.5–0.75	256	8	32	1	0.25
*C. albicans* ATCC 10231	128	4	64	1	0.75	512	8	64	1	0.25

MIC, minimum inhibitory concentration; MBC, minimum bactericidal concentration; MIC’, the MIC of allicin in combination with domiphen; MBC’, the MBC of allicin in combination with domiphen; FIC, The fractional inhibitory concentration; FBC, The fractional bactericidal concentration. FIC > 0.5, no synergistic effect was observed, whereas FBC < 0.5 indicates a synergistic effect.

The effectiveness of combining allicin with domiphen against *S. aureus* ATCC 6538, *E. coli* 8099, and *C. albicans* ATCC 10231 was evaluated using the checkerboard assay. The MBC values for *E. coli*, *S. aureus*, and *C. albicans* were (32 + 1) μg/mL, (64 + 1/2) μg/mL, and (64 + 1/2) μg/mL, respectively, when allicin was combined with domiphen (Table 1). The FIC values were greater than 0.5, and the FBC values were 0.38, 0.25, and 0.25 for *S. aureus*, *E. coli*, and *C. albicans*, respectively. These results suggest that the combination of allicin and domiphen has a synergistic bactericidal effect.

### 3.2 The effect of allicin or domiphen against the biofilm

**Figure 1:**
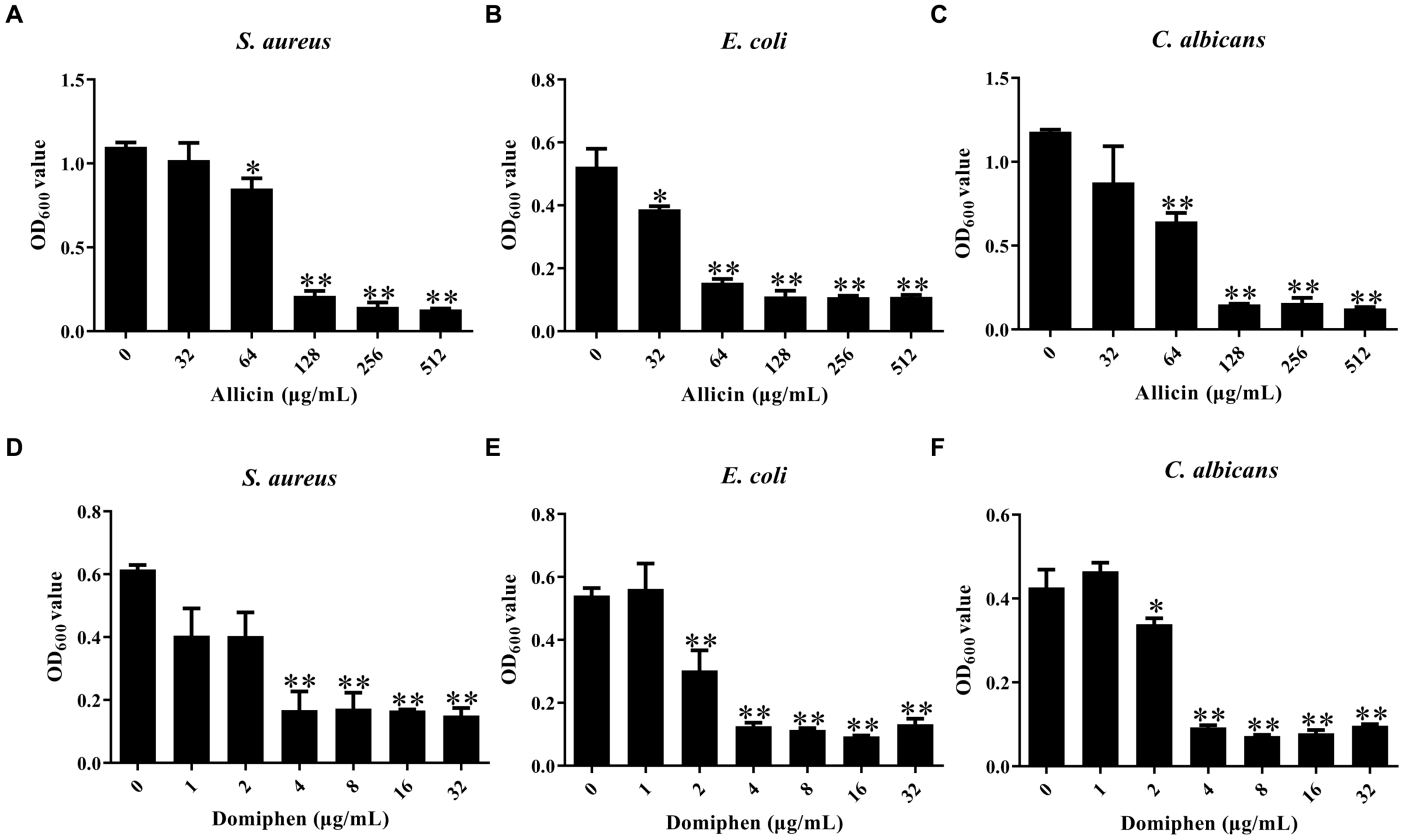
The effect of allicin or domiphen against the microbial biofilm. The inhibitory effect of allicin against biofilms of *S. aureus* **(A)**, *E. coli* **(B)**, and *C. albicans* **(C)**. The inhibitory effect of domiphen against biofilms of *S. aureus* **(D)**, *E. coli* **(E)**, and *C. albicans* **(F)**. Data are presented as means ± standard deviation. Compared to the control group, * indicates *p* < 0.05, and ** indicates *p* < 0.01.

To investigate the inhibitory effect of allicin and domiphen against the biofilm of *S. aureus*, *E. coli*, and *C. albicans*, the 96-well plate crystal violet staining method was used. Compared to the control group, 64 μg/mL allicin was effective in *S. aureus* and *E. coli* biofilm removal (Figures 1A,B, *p* < 0.01), and 32 μg/mL allicin effectively decreased the biofilm of *C. albicans* (Figure 1C, *p* < 0.05). Similarly, 4 μg/mL domiphen significantly decreased the biofilm of *S. aureus* (Figure 1D, *p* < 0.01). Additionally, 2 μg/mL domiphen visibly reduced the biofilm of *E. coli* (Figure 1E, *p* < 0.01) and *C. albicans* strains (*p* < 0.05).

After treatment with allicin, the MBEC_50_ values for *S. aureus*, *E. coli*, and *C. albicans* were 128 μg/mL, 64 μg/mL, and 128 μg/mL, respectively. This suggests that allicin treatment was more effective in reducing biofilm formation by the *E. coli* than that by the other two strains. The MBEC_50_ values of domiphen were 4 μg/mL for all three strains (Figure 1F), indicating that domiphen was equally effective in biofilm removal for the three pathogens.

**Figure 2:**
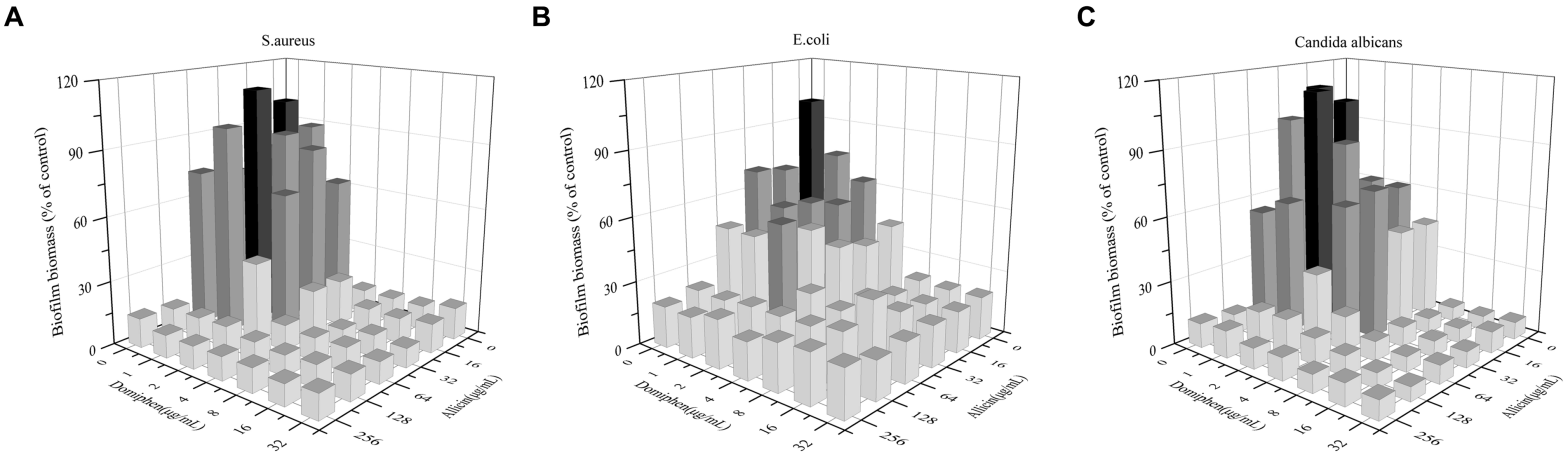
*S. aureus*, *E. coli*, and *C. albicans* biofilm removal using a drug combination. Combination of allicin and domiphen against biofilms of *S. aureus* **(A)**, *E. coli* **(B)**, and *C. albicans* **(C)** strains. Dark represents the control group, dark gray represents clearances ranging from 50 to 99%, and light gray indicates clearance below 50%.

The effectiveness of the combination of allicin and domiphen for biofilm removal was further investigated. The MBEC_50_ values of allicin combined with domiphen was (32 + 1) μg/mL against *E. coli* and *C. albicans* strains (Table 2 and Figure 2). The *E. coli* biofilm removal was significantly improved to approximately 53.44% (*p* < 0.01, Figure 2B), whereas that of *C. albicans* strains was 50.95% (*p* < 0.01, Figure 2C). Similarly, 64 μg/mL allicin combined with 1 μg/mL domiphen decreased *S. aureus* biofilm by approximately 67.99% (*p* < 0.01, Figure 2A). The fractional biofilm eradication concentration (FBEC) of allicin combined with domiphen against *S. aureus* and *E. coli* biofilms was 0.75, indicating an additive effect. The FBEC value for *C. albicans* biofilm was 0.5, indicating a synergistic interaction (Table 2).

**Table 2:** Minimum biofilm-eliminating concentration of 50%.

Strains	MBEC_50_ (μg/mL)		MBEC_50_’ (μg/mL)		FBEC
	Allicin	Domiphen	Allicin	Domiphen	
*S. aureus* ATCC 6538	128	4	64	1	0.75
*E. coli* 8099	64	4	32	1	0.75
*C. albicans* ATCC 10231	128	4	32	1	0.5

MBEC_50_, minimum biofilm eradication concentration that can eliminate 50% of the pathogens in pre-formed biofilms; MBEC_50_’, the MBEC_50_ of allicin in combination with domiphen; FBEC, fractional biofilm eradication concentration. FBEC ≤ 0.5 indicates a synergistic effect between the two drugs, FBEC > 0.5 to 1 indicates an additive effect, FBEC > 1 to < 2 indicates no difference, and ≥ 2 indicates antagonism.

### 3.3 The combination of allicin and domiphen removes biofilm on stainless steel surface

**Figure 3:**
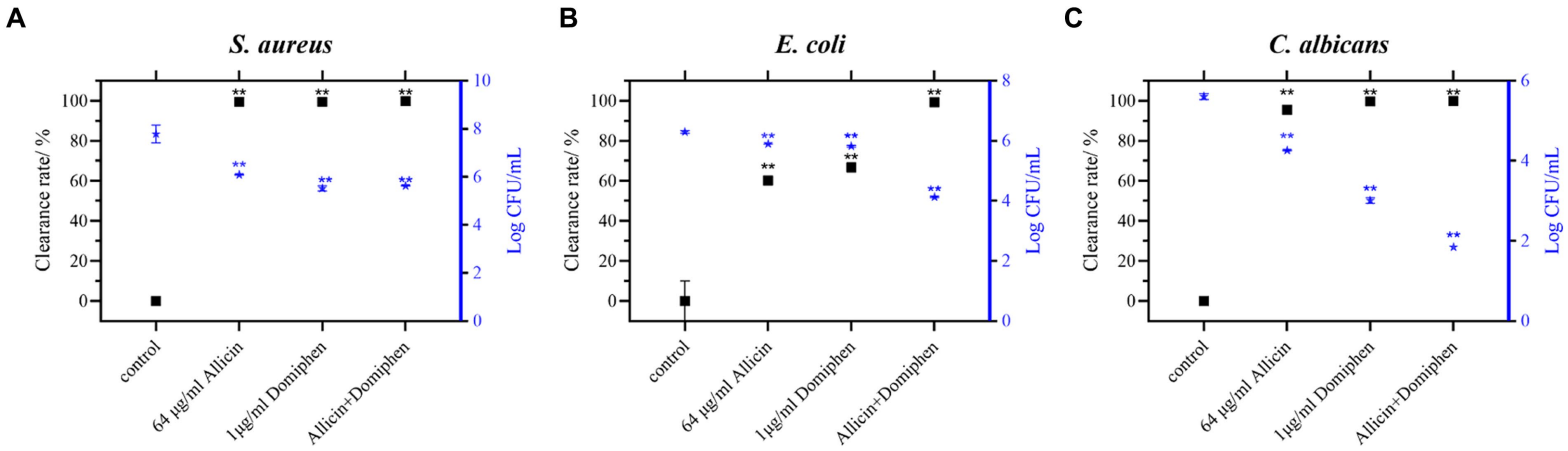
Combination of allicin and domiphen to remove biofilms on stainless steel surfaces. Viable cells of *S. aureus* **(A)**, *E. coli* **(B)**, and *C. albicans* **(C)** strains on stainless-steel surfaces after treatment with a combination of allicin and domiphen. Data are presented as means ± standard deviation. Compared to the control group, ** indicates *p* < 0.01, * indicates *p* < 0.05.

*Staphylococcus aureus*, *Escherichia coli*, and *Candida albicans* were selected to investigate the effect of allicin combined with domiphen on biofilm removal from stainless steel surfaces (Figure 3). Compared to the control group, the combination of 64 μg/mL allicin and 1 μg/mL domiphen significantly removed the biofilms of *S. aureus* ATCC 6538 (Figure 3A) and *E. coli* 8099 (Figure 3B) and reduced the total viable count of biofilm cells by more than 10^2^ CFU/mL (*p* < 0.01). The total viable count of *E. coli* biofilm bacteria using allicin (64 μg/mL) or domiphen (1 μg/mL) alone was reduced by less than 10-fold, and the difference was not significant. In addition, the combination of 64 μg/mL allicin and 1 μg/mL domiphen significantly removed *E. coli* biofilm (*p* < 0.01) and reduced the total viable count of biofilm bacteria by more than 10^2^ CFU/mL.

Compared with the control, allicin combined with domiphen significantly removed *C. albicans* ATCC 10231 biofilm (Figure 3C, *p* < 0.01) and reduced the total viable count of biofilm cells by approximately 10^3^ CFU/mL. However, the total viable count of *C. albicans* biofilm cells using allicin (64 μg/mL) or domiphen (1 μg/mL) alone was reduced by less than 10^2^ CFU/mL (*p* < 0.01). Notably, the biofilm removal was significantly enhanced by the combination of the two drug (*p* < 0.01).

### 3.4 The combination of allicin and domiphen removes biofilm on polyethylene surfaces

**Figure 4:**
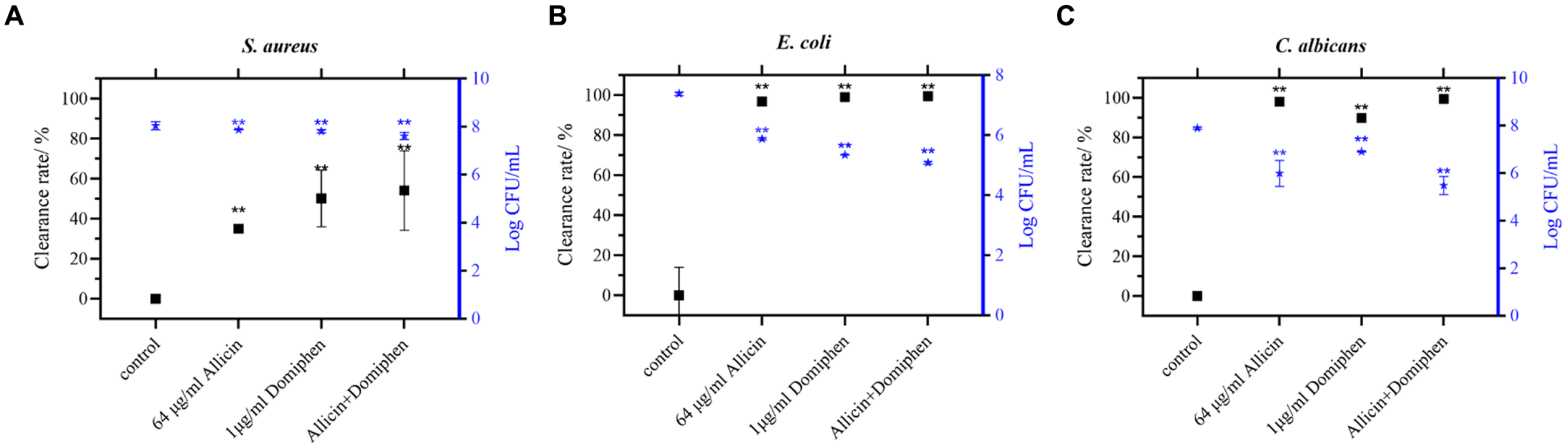
Combination of allicin and domiphen to remove biofilms on polyethylene material surfaces. Viable cells of *S. aureus* **(A)**, *E. coli* **(B)**, and *C. albicans* **(C)** strains on polyethylene material surfaces after treatment with a combination of allicin and domiphen. Data are presented as means ± standard deviation. Compared to the control group, ** indicates *p* < 0.01, * indicates *p* < 0.05.

On the polyethylene surfaces (Figure 4), the biofilms of *E. coli* 8099 (Figure 4B) and *C. albicans* ATCC 10231 (Figure 4C) were significantly removed using the drug combination (*p* < 0.01), and the total viable count of biofilm cells was reduced by more than 10^2^ CFU/mL. Compared to the control group, the total viable count of *E. coli* or *C. albicans* biofilm cells using allicin (64 μg/mL) or domiphen (1 μg/mL) alone was reduced approximately 10-fold (*p* < 0.01). Additionally, the drug combinations significantly increased the removal of *S. aureus* biofilm (Figure 4A, *p* < 0.01). Overall, the drug combinations exhibited a higher efficacy for biofilm removal.

### 3.5 Biofilm removal by drug combination

**Figure 5:**
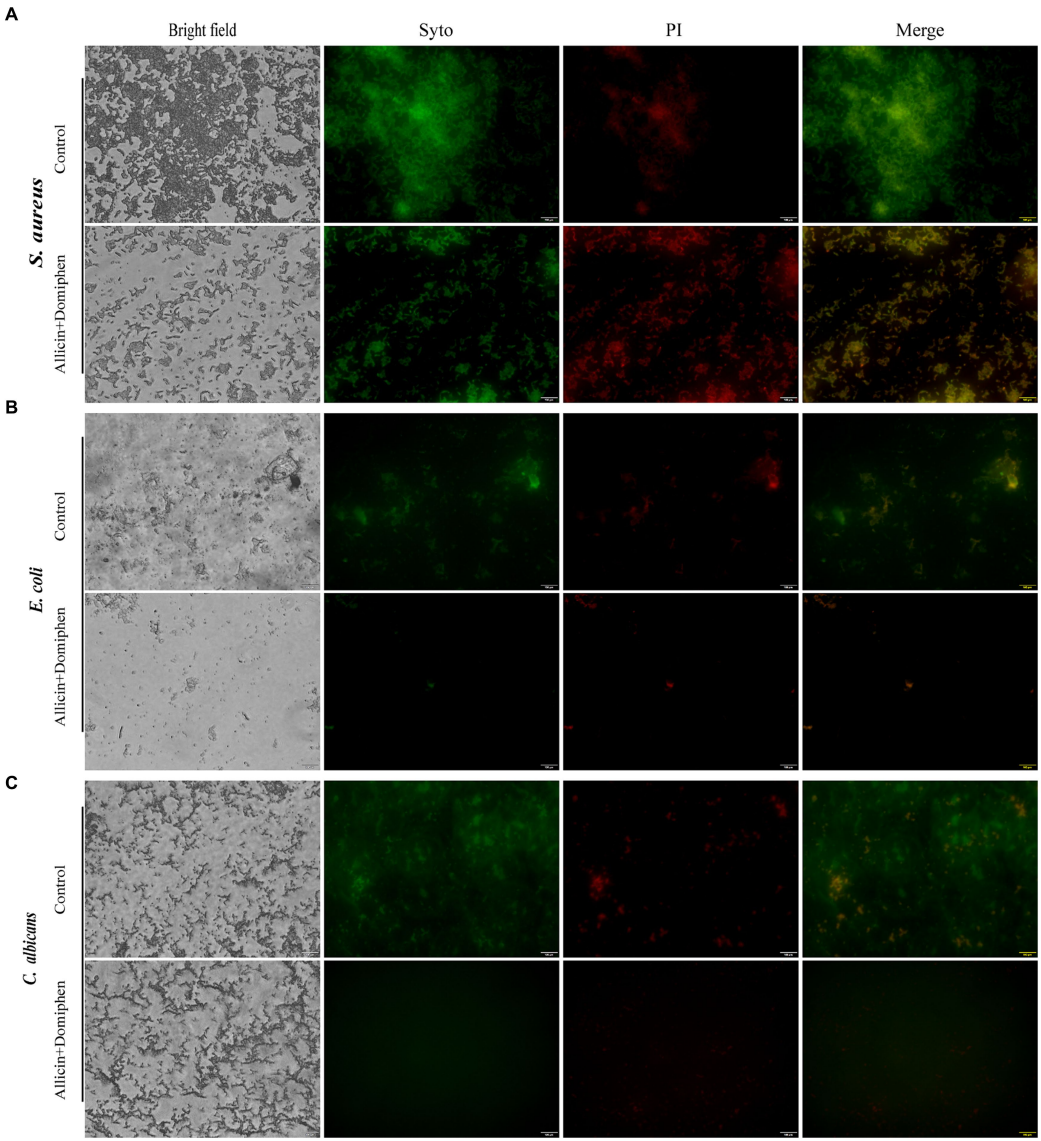
Characterization of microbial biofilm removal using allicin combined with domiphen. FM images of *S. aureus* ATCC 6538 **(A)**, *E. coli* 8099 **(B)**, and *C. albicans* ATCC 10231 **(C)** biofilms with the drug combination. Control: Microbial biofilms treated with phosphate buffer solution. Images correspond to bright-field, SYTO9 (living cells with green fluorescent), PI (dead cells with red fluorescence), and merged image of the microbial biofilm. Bar = 100 μm.

The control group was not treated with any drug. Dense and thick biofilms of *S. aureus* ATCC 6538 (Figure 5A), *E. coli* 8099 (Figure 5B), and *C. albicans* ATCC 10231 (Figure 5C) exhibited green fluorescence. Biofilms of *S. aureus*, *E. coli*, and *C. albicans* were effectively eliminated by treatment with allicin combined with domiphen. The cells in the biofilms of the three strains treated with the drug combination were killed, and the microorganisms appeared noticeably thinner and more dispersed. Dead bacteria were easily observed with red fluorescence. Furthermore, the biofilm matrices of the three strains containing the drug combination diffused to the contact surface.

## 4 Discussion

*Staphylococcus aureus*, *Escherichia coli*, and *Candida albicans* are opportunistic pathogens that cause challenging clinical diseases. These microorganisms are prone to forming biofilms and developing drug resistance under harsh environmental conditions, posing a serious threat to human health. According to the World Health Organization, drug-resistant diseases contribute to a global mortality rate of approximately 700,000 individuals (Liu et al., 2022). Further studies are required to identify agents that can reduce biofilm formation and decrease drug resistance.

Studies have shown that allicin inhibits pathogenic bacteria such as *Porphyromonas gingivalis* (Bachrach et al., 2011), *Streptococcus mutans* (Bachrach et al., 2011; Lee et al., 2011), *Actinomyces viscosus* (Bachrach et al., 2011), and *Lactobacillus acidophilus* (Lee et al., 2011), whereas domiphen can inhibit *E. coli* and *C. albicans* (Tits et al., 2020b; Hu et al., 2021). The antimicrobial effects of allicin and domiphen against *S. aureus*, *E. coli*, and *C. albicans* was investigated. The MIC value of allicin against the three strains was 128 μg/mL according to the results (Table 1). Similarly, the MIC value of allicin against *Enterobacter cloacae* was reported to be 125 μg/mL (Tao et al., 2023). Notably, the MIC value of allicin against *S. mutans*, *A. viscosus*, and *A. israelii* was found to be 600 μg/mL (Bachrach et al., 2011). The combination of allicin and domiphen significantly reduced the dosage of both agents against the three pathogens compared to single-agent application, and the FBC indices indicated that the bactericidal effects were synergistic. This suggests that these two drugs can be used in combination to disinfect contact surfaces in food industry. The results of susceptibility testing for drug combinations are reproducible and necessary for practical application. Natural products (EOs) have multi-target inhibitory effects on pathogens, combining them with agents can enhance the activity of the drugs and avoid the emergence of microbial drug resistance (Ju et al., 2020). More time and experiments are needed to determine whether the combination of allicin and domiphen can avoid resistance due to continuous use. The scale-up of gram-negative bacteria, gram-positive bacteria, and fungal that treatment with drug combination are potential in the future.

The cost of drug reformulation is significantly lower than that of developing a new drug (De Cremer et al., 2015). Combining an existing antimicrobial drug with another compound provides a synergistic effect on antimicrobial and antifungal biofilms, and is a highly effective therapeutic approach. Moreover, combining miconazole with domiphen (Tits et al., 2020b), or allicin with ciprofloxacin have synergistic anti-biofilm effects (Bhattacharya et al., 2022). To reduce the use of antibiotics, with the combination of allicin and domiphen was selected as an effective antibiotic-free strategy (Figure 2). The combination of these drugs exhibited a synergistic effect against *C. albicans* biofilms. A previous study investigated the effects of allicin on the adhesion ability, biofilm formation, swimming motility, and dispersal of uropathogenic *E. coli* (UPEC) CFT073 and J96. Allicin reduced UPEC biofilm formation, altered its structure, and dispersed the biofilms (Yang et al., 2016). In another study, domiphen increased the permeability vacuolar membranes of *Candida* spp. (Tits et al., 2020a). Domiphen enhances the distribution of beneficial agents within the biofilm matrix, leading to the accumulation of reactive oxygen species and enhanced damage to pathogenic cells (Tits et al., 2020a,b). It was suggested that the combination of allicin and domiphen reduced biofilm adhesion, increased drug permeability, damaged the biofilm bacteria and inhibited biofilm formation.

In addition, the antimicrobial and anti-biofilm effects of the drug against pathogens on stainless steel (Figure 3) and polyethylene surfaces (Figure 4) were investigated. Polyethylene (Cheng et al., 2015) and stainless steel (Vreuls et al., 2010; Yang and Ren, 2010) are extensively used in medical device fabrication and food packaging, exert a substantial influence on our daily lives (Lambré et al., 2022). The combination of 64 μg/mL allicin and 1 μg/mL domiphen reduced the total viable count of *E. coli* and *C. albicans* biofilm cells by more than 10^2^ CFU/mL. Biofilm formation of the three pathogens was different on stainless steel surface. The potential variability of biofilm reactions due to differences in microbial strains and environmental conditions. Microorganisms evolved to be specialists in biofilm formation as a part of the adaptive defensive strategy, providing protection from adverse environmental stresses and antibiotic treatment (Chu et al., 2018). The formation of these microbial cells and their eventual dispersal is commanded through diverse and specific sophisticated mechanisms (McDougald et al., 2011). Anti-biofilm activity was evaluated using fluorescence microscopy, which revealed that the combination of allicin and domiphen was effective in diffusing *S. aureus*, *E. coli*, and *C. albicans* biofilms, similar to other reported drug combinations (Bezerra et al., 2022). In addition, rhamnolipid, a surfactant similar to domiphen, has been shown to disperse biofilms and develop antibacterial and anti-biofilm properties (Khalid et al., 2019). Similarly, domiphen disperses in the microbial biofilm matrix, inhibits the adhesion of pathogenic cells, increases the antimicrobial effect of the drug combination, and prevents repeated contamination. These results suggest that the combination of allicin and domiphen can be applied in the food and medical fields, specifically in packaging and processing equipment made of polyethylene or stainless steel.

## 5 Conclusion

In summary, both allicin and domiphen exhibited antimicrobial effects. Additionally, the combination of allicin and domiphen demonstrated improved antimicrobial and anti-biofilm properties. This provides a promising option for developing novel drug combination suitable for antimicrobial and biofilm dispersions on polyethylene and stainless steel surfaces.

## Data availability statement

The original contributions presented in the study are included in the article/supplementary material, further inquiries can be directed to the corresponding authors.

## Author contributions

SL: Methodology, Writing – original draft. YW: Methodology, Validation, Writing – original draft. GX: Conceptualization, Software, Writing – review & editing. YX: Data curation, Writing – review & editing. CF: Methodology, Writing – review & editing. QZ: Data curation, Writing – review & editing. LX: Methodology, Writing – review & editing. XJ: Data curation, Writing – review & editing. YZ: Data curation, Writing – review & editing. YL: Data curation, Writing – original draft, Writing – review & editing. JQ: Data curation, Writing – original draft, Writing – review & editing.
